# A Novel UPLC-MS/MS Assay for the Measurement of Linezolid and its Metabolite PNU-142300 in Human Serum and its Application to Patients With Renal Insufficiency

**DOI:** 10.3389/fphar.2021.641872

**Published:** 2021-05-20

**Authors:** Yingying Wang, Er-min Gu, Xiaoxiang Du, Ren-ai Xu, Guanyang Lin

**Affiliations:** ^1^ Department of pharmacy, The First Affiliated Hospital of Wenzhou Medical University, Wenzhou, China; ^2^ The First People’s Hospital of Jiashan, Jiaxing, China

**Keywords:** linezolid, PNU-142300, myelosuppression, human serum, UPLC-MS/MS

## Abstract

The contribution of the metabolites of linezolid to the associated myelosuppression is unknown in patients who are renal impairment. In this research, the purpose of our experiment was to explore and develop a quick and robust ultra performance liquid chromatography tandem mass spectrometry (UPLC-MS/MS) assay for the determination of linezolid and its metabolite PNU-142300 in human serum simultaneously. The analytes were prepared using a simple and convenient approach with acetonitrile for protein crash, and then separated from the matrix on a Waters Acquity Ultra performance liquid chromatography (UPLC) BEH C18 (2.1 mm × 50 mm, 1.7 μm) column in a program of gradient elution, where the mobile phase was consisted of water with 0.1% formic acid and acetonitrile, and was placed at 0.40 ml/min flow rate. Multiple reaction monitoring (MRM) was employed and conducted for UPLC-MS/MS detection with ion transitions at *m/z* 338.01 → 296.03 for linezolid, *m/z* 369.96 → 327.98 for PNU-142300 and *m/z* 370.98 → 342.99 for tedizolid (Internal standard, IS), respectively. This method had good linearity respectively in the calibration range of 0.01–20 μg/ml for linezolid, and 0.05–100 μg/ml for PNU-142300. In the intra- and inter-day, the precision of linezolid and PNU-142300 was below 14.2%, and the accuracy in this method was determined to be from −9.7 to 12.8%. In addition, recovery and matrix effect of the analytes were all found to be acceptable, and the analytes during the assay and storage in serum samples were observed to be stable. The novel optimized UPLC-MS/MS assay was also successfully employed to determine the concentration levels of linezolid and PNU-142300 in human serum. The results showed that linezolid-associated myelosuppression occurs more frequently in patients with renal insufficiency, and the metabolite-to-parent concentration ratio of PNU-142300 is predicted to reduce this toxicity of myelosuppression.

## Introduction

**FIGURE 1:**
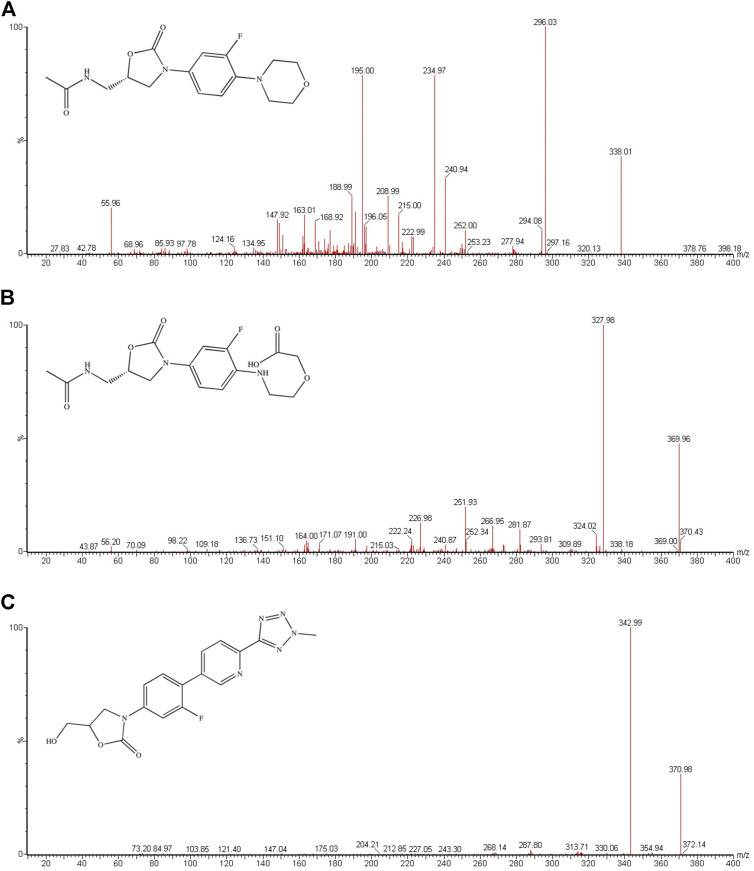
Mass spectra of linezolid **(A)**, PNU-142300 **(B)** and tedizolid (IS, **(C)** in this study.

Severe infection is raising a serious and major problem of health in this world, and is increasing the morbidity and mortality of patients in the intensive care unit (ICU) (Fleischmann et al., 2016). Linezolid (Figure 1A), an oxazolidinone antibiotic, was approved for the therapy of infections in 2000, which were caused by Gram-positive resistant bacteria, such as complicated and uncomplicated skin infections, Methicillin-resistant *Staphylococcus aureus* (MRSA), vancomycin-resistant enterococci, community-acquired pneumonia and nosocomial pneumonia (Hashemian et al., 2018). Linezolid is usually based on standard dosing protocols, and the eliminated dose with unchanged form in the urine is about 30–40%. However, linezolid-associated myelosuppression limits its use, because the time for the treatment of complicated infections required >14 days (Kato et al., 2021). In addition, numerous studies pointed out that linezolid-associated myelosuppression is associated with the trough concentrations of linezolid, and the risk of this toxicity could be reduced by therapeutic drug monitoring (TDM) (Cattaneo et al., 2016; Galar et al., 2017; Pea et al., 2017; Rao et al., 2020). Moreover, from real-world clinical researches, renal function has been considered as a significant risk factor of interpatient variability in linezolid trough concentrations (Morata et al., 2016; Crass et al., 2019).

Through poorly described oxidation pathways, linezolid is primarily metabolized into PNU-142300 (Figure 1B) and PNU-142586, and both of them exceeded 10% of the dose of the parent drug excreted in the urine, with specific value of 10 and 40% respectively (Stalker and Jungbluth, 2003). Although it has been reported that renal function is not an important influencing factor of the overall exposure of linezolid in plasma, the two main metabolic products in patients with renal insufficiency have been identified and determined to be higher than normal patients (Stalker and Jungbluth, 2003; Souza et al., 2020a; Souza et al., 2020b). Therefore, it is urgent to validate and establish a bioanalytical assay for the simultaneous measurement of linezolid, PNU-142300, and PNU-142586 in serum to investigate the contribution of each analyte to toxicity in patients with different renal function.

Until now, there were only two literatures reported for simultaneous measurement of linezolid, and its two main metabolites (PNU-142300 and PNU-142586) in serum from human. Ultra performance liquid chromatography (UPLC) with ultraviolet-visible spectroscopy was the first published approach, and the disadvantages of this assay have small number of clinical samples, long chromatographic retention time and low sensitivity (Sakurai et al., 2019). Recently, another analytical approach based on liquid chromatography tandem mass spectrometry (LC-MS/MS) was established with the concentration range of 0.1–50 μg/ml for linezolid and PNU-142300, and 0.1–25 μg/ml for PNU-142586, which has limited availability of isotope-labeled internal standard (IS) and rather long run time (Souza et al., 2020b). Therefore, there is no bioanalytical method to satisfy the accurate and high-throughput detection of linezolid, along with its two major metabolites in clinical samples.

The two metabolites (PNU-142300 and PNU-142586) are not bought available, and we only obtained PNU-142300 from WuXi AppTec. Thus, the purpose of the current work was to establish a simple and accurate ultra performance liquid chromatography tandem mass spectrometry (UPLC-MS/MS) assay for simultaneously quantifying the concentration levels of linezolid and PNU-142300 in human serum and to investigate the effect of renal function on the concentration levels of linezolid and PNU-142300 to support future personalized medicine by TDM strategies targeted to the reduction of the risk of linezolid-associated myelosuppression toxicity.

## Experimental method

### Chemicals Materials

The purity of linezolid and tedizolid (used as internal standard, IS, Figure 1C) were >98%, and were offered from Beijing sunflower and technology development CO., LTD. (Beijing, China). PNU-142300 (purity >98%) was synthesized from WuXi AppTec (Jiangsu, China). Methanol and acetonitrile in this study were LC grade, and both of them were supplied by Merck Company (Darmstadt, Germany). Beijing sunflower and technology development CO., LTD. (Beijing, China) also provided formic acid, which was analytical grade. In addition, ultrapure water was made by a Water Purification System from Milli-Q (Millipore, Bedford, United States) to meet the need of the whole experiment.

### Ultra Performance Liquid Chromatography Tandem Mass Spectrometry Conditions

Liquid chromatographic analysis was carried out by a Waters ACQUITY UPLC I-Class system (Milford, MA, United States) fitted with a Waters Xevo TQ-S triple quadrupole tandem mass spectrometer equipped with an electrospray ionization (ESI) source (Milford, MA, United States). Masslynx 4.1 software and Quanlynx programme (Milford, MA, United States) in this experiment were used to acquire and process the data of this whole experiment and control the instruments.

The UPLC experiments on the chromatographic separation of linezolid, PNU-142300 and IS was performed on an Acquity UPLC BEH C18 column (2.1 mm × 50 mm, 1.7 μm). In addition, the mobile phase in this study, consisting of solvent A (acetonitrile) and solvent B (0.1% formic acid in water), was employed to separate the analyte and IS using a linear gradient program with a flow rate of 0.40 ml/min as follows: 10–10% A (0–0.5 min), 10–90% A (0.5–1.0 min), 90–90% A (1.0–2.0 min), and 90–10% A (2.0–2.1 min). Then, the post time was 0.9 min. The injection volume of each needle was 0.2 µl, and the entire time for each analysis was about 3.0 min. The autosampler was set at 10°C for each sample analysis, and the column temperature was maintained at 40°C temperature.

Detection was finished for the measurement of experimental samples in the positive ion mode through an ACQUITY UPLC system equipped with a Xevo TQ-S triple quadrupole tandem mass spectrometer. Multiple reaction monitoring (MRM) method was selected to detect the analytes and IS in serum, in which the precursor-to-product ion transitions for linezolid, PNU-142300 and IS were *m/z* 338.01 → 296.03, *m/z* 369.96 → 327.98, and *m/z* 370.98 → 342.99, respectively. The collision energy and cone voltage were respectively 15 eV and 30 V for linezolid, PNU-142300 and IS. The general MS parameters were optimized as follows: desolvation temperature 600°C, capillary voltage 2.0 kV, collision gas 0.15 ml/min, cone gas 200 L/h, and desolvation gas 1000 L/h.

### Preparation of Stock Solutions, Calibration Standards, and Control Samples

Each stock solution of linezolid, PNU-142300 and IS at the corresponding concentration level of 1.00 mg/ml was individually obtained by dissolving each compound into an accurate amount of methanol for quality control (QC) and calibration curve samples. A mixed-standard working solutions were prepared by series dilution of the stock solution of each analyte with methanol. Then, calibration curves spiked with blank human serum were operated by ten-fold dilution of the mixed-standard working solutions in order to get final concentration levels in the calibration curve range of 0.01–20 μg/ml for linezolid, and 0.05–100 μg/ml for PNU-142300, respectively. Similary, QC samples at LLOQ (lower limit of quantification), low (LQC), medium (MQC) and high (HQC) concentration levels were also prepared as follows: 0.01, 0.02, 2.0, 16 μg/ml for linezolid, and 0.05, 0.1, 10, 80 μg/ml for PNU-142300, respectively. The IS working solution was obtained by diluting the stock solution with methanol to produce the required concentration of 5.0 μg/ml. All prepared solutions in this experiment including the stock and working solutions were ready in advance and placed at −40°C for further use.

### Preparation of Samples

All the samples in our study were processed by using a simple and rapid protein precipitation approach with acetonitrile. To each serum sample with a volume of 100 µl in 1.5 ml EP centrifuge tube, 20 µl IS working solution at the concentration level of 5.0 μg/ml was spiked and mixed for 0.5 min. Then, plasma proteins were immediately precipitated by adding 300 µl of acetonitrile, and the mixture was vigorously vortexed for 1.0 min, and centrifugated at 13,000 g for 10 min at room temperature. Finally, 100 µl aliquot of the clear supernatant was used to transfer into the new auto-sampler vial, and 0.2 µl of the solution was added into the UPLC-MS/MS system for quantitative determination and data acquisition.

### Method Validation

Fully method validation procedures for this analytical method, including selectivity, the lineary of calibration curve, LLOQ, precision and accuracy, recovery, matrix effect, and stability, were conducted according to the FDA principles on the validation of the bioanalytical assay (Xu et al., 2019; Tang et al., 2020).

In this experiment, the selectivity of this method was investigated by checking the absence of endogenous interferences from the blank (neither analyte nor IS from six different human samples), standard solutions and real human serum at the corresponding retention time of the respective analyte of linezolid, PNU-142300, and IS, as previously described (Qiu et al., 2019).

Calibration curves of the analytes were evaluated by describing the relationship between the peak area ratio of each analyte to IS and the nominal concentrations of each analyte by least square regression mode with a 1/*x* ^2^ weighting factor. The sensitivity of this assay was performed in terms of LLOQ, which was identified as the lowest point on the calibration curve, and should be investigated with precision below 20% and accuracy within ±20% of the nominal value.

Precision and accuracy of the analytes in the experiment were estimated by sextuple detection of three concentration levels of QC samples over three consecutive days. Relative standard deviation (RSD, %) stands for precision, and relative error (RE, %) stands for accuracy. Recovery from present approach of sample preparation was investigated by comparing the peak area ratio of each analyte before and after the extraction, respectively. Matrix effect (ME) was also analyzed in 6 replicates through the comparison of the response of each substance in serum matrix after extraction with that in neat solution.

The stability of each analyte in spiked serum was examined by detecting LQC and HQC samples under different storage conditions. Short-term stability was estimated at room temperature for 4 h, and long-term stability was evaluated at −40°C for 3 weeks. In addition, post-preparation stability was detected for QC samples placed in the autosampler at 10°C for 8 h. Moreover, three complete freez/thaw stability (−40°C to room temperature) was also studied.

### Application in Clinical Samples

The established UPLC-MS/MS bioanalytical assay was employed to determine simultaneously the serum concentration levels of linezolid and PNU-142300 in 115 samples from 89 patients who were treated with oral or intravenous linezolid at The First Affiliated Hospital of Wenzhou Medical University. The patients were segregated into five different groups according to the Chronic Kidney Disease Epidemiology Collaboration (CKD-EPI) equation based on estimated glomerular filtration rate (eGFR, mL/min/1.73 m^2^): patients with eGFR≥90 (Group A, healthy), 60 ≤ eGFR < 90 (Group B, mild impaired), 30 ≤ eGFR < 60 (Group C, moderate impaired), eGFR < 30 (Group D, severe impaired), and continuous renal replacement therapy (CRRT, Group E, end-stage renal disease, ESRD). All patients were sampled at least after the 7th dose under steady state conditions. The serum samples of the analytes were collected, followed by centrifugation at 4,000 × g for 8 min at room temperature, and then immediately placed at −80°C until further analysis. The protocol of our study was assessed and approved by the Ethics Committee of The First Affiliated Hospital of Wenzhou Medical University (No. 2020-zz-019).

### Statistical Methods

In our study, Statistical Package for the Social Sciences version 19.0 (SPSS, Chicago, IL, United States) software was used to calculate the results of all statistical analysis, and *P* < 0.05 was considered as meaningful in statistical analysis. Meanwhile, receiver operating characteristic (ROC) curve and its associated areas under the curve were used to assess the predictive power of the metabolite-to-parent concentration ratios for linezolid-associated myelosuppression; meanwhile, the cut-off point with the highest discriminative ability was chosen based on the maximum Youden’s index.

## Results and Discussion

### Method Development and Optimization

During method development, chromatographic conditions under different environments were investigated and assessed in order to get good results of the separation, peak shape, and mass response. In our study, five kinds of the solutions (including methanol, acetonitrile, water, ammonium acetate and formic acid), were evaluated, and it was found that the sensitivity was enhanced and the peak shape was improved when formic acid was added to aqueous phase. Thus, in our present experiment, acetonitrile as organic phase and 0.1% formic acid in water as aqueous phase were selected to offer higher intensity, lower background noise, and better peak shapes.

Given the complex nature of serum, a procedure for removing potential interferents and proteins in sample pretreatment is often required before LC-MS/MS analysis. Protein precipitation (PPT), liquid-liquid extraction (LLE) and solid-phase extraction (SPE) are the most common methods applied to the preparation of the biological samples (Gregoire et al., 2016; Hedaya et al., 2017; Paal et al., 2018; Ferrari et al., 2019). Compared with PPT for sample pretreatment, SPE is relatively time consuming and expensive, and LLE is relatively complicated. Thus, PPT method was adopted and investigated at the beginning of the method development. It was found that extraction recovery of each analyte was within the acceptable range, and no matrix effect was observed. Thus, in this present experiment, PPT method was adopted for it offered many advantages, such as low cost, quick and simple operation, and enough extraction recovery rates in the sample pretreatment.

### Method Validation in the Current Study

#### Selectivity

**FIGURE 2:**
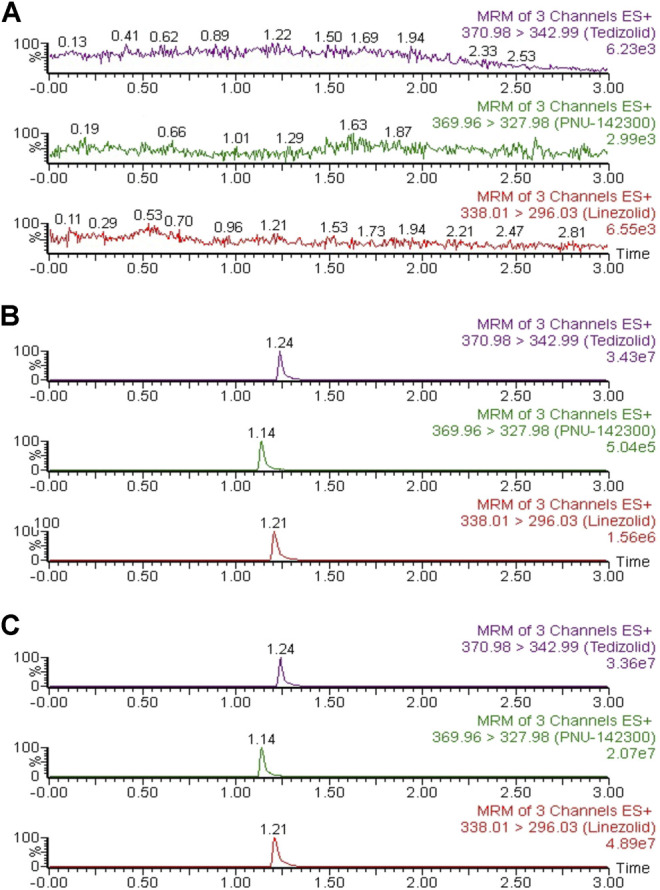
Representative chromatograms of linezolid, PNU-142300, and IS in human serum: **(A)** blank serum; **(B)** blank serum spiked standard solutions (1.0 μg/ml for linezolid and IS, 2.0 μg/ml for PNU-142300); **(C)** a real human serum.

As indicated in Figure 2, the accurate retention times of linezolid, PNU-142300 and IS in this study were respectively 1.21, 1.14 and 1.24 min. Comparison of the representative MRM chromatograms of blank human serum from 6 individual sources, blank serum added with standard solutions and IS, and the actual serum sample demonstrated that no potential interfering substances was found. It suggested that this method had a good selectivity to determine linezolid, PNU-142300 and IS in human serum.

#### Calibration Curve and Lower Limit of Quantification

At the concentration ranges of 0.01–20 μg/ml for linezolid, and 0.05–100 μg/ml for PNU-142300 in the calibration curve, the representative linear regression equations of peak ratios (Y) vs. the matching concentrations (X) were as follows: *Y* = 3.50032 × X ± 0.0701,292 (*r* ^2^ = 0.9985) for linezolid, and *Y* = 0.197,551 × X ± 0.0441,901 (*r* ^2^ = 0.9978) for PNU-142300, both of which exhibited excellent linearities. LLOQ was identified as the sensitivity in this assay, and was detected as 0.01 μg/ml for linezolid and 0.05 μg/ml for PNU-142300 respectively with acceptable accuracy and precision validated by the bioanalysis guidelines (Table 1) (Mei et al., 2019).

**TABLE 1:** The precision and accuracy of linezolid and PNU-142300 in human serum (*n* = 6).

Analytes	Concentration (µg/ml)	Intra-day		Inter-day	
		RSD%	RE%	RSD%	RE%
Linezolid	0.01	9.2	4.5	10.4	5.4
	0.02	14.2	2.3	8.1	12.8
	2.0	5.9	6.2	4.4	11.6
	16	0.7	−9.7	1.9	−9.7
	0.05	9.5	7.3	11.1	−3.9
PNU-142300	0.1	7.2	−1.0	8.3	9.6
	10	5.1	−8.3	7.7	0.6
	80	2.8	3.1	2.7	3.3

#### Accuracy and Precision

The precision and accuracy of the developed UPLC-MS/MS assay were calculated by detecting QC samples at HQC, MQC, LQC and LLOQ four various concentration levels over three different days (*n* = 6). As listed in Table 1, the results of the accuracy were determined to be ranged from −9.7 to 12.8%, and the intra-day and inter-day of precision was <14.2% and <11.1%, respectively. These data indicated that the described UPLC-MS/MS bioanalytical assay provided suitable accuracy and precision for the simultaneous quantification of linezolid and PNU-142300 in human serum (Nyssen et al., 2016).

#### Matrix Effect and Recovery

At LQC, MQC and HQC three different concentration levels, the data of matrix effect and recovery were presented in Table 2. The mean recoveries of the analytes were within the range of 85.7–96.8%, and the matrix effect values of the analytes were 99.9–108.3%, suggesting that there were no significant matrix effects in human serum using the present optimized UPLC-MS/MS assay for analysis.

**TABLE 2:** Recovery and matrix effect of linezolid and PNU-142300 in human serum (*n* = 6).

Analytes	Concentration (µg/ml)	Recovery (%)		Matrix effect (%)	
		Mean ± SD	RSD (%)	Mean ± SD	RSD (%)
Linezolid	0.02	85.7 ± 12.0	14.0	99.9 ± 11.8	11.8
	2.0	89.0 ± 4.9	5.5	108.3 ± 7.6	7.1
	16	96.2 ± 4.5	4.6	101.6 ± 3.4	3.4
	0.1	91.5 ± 11.1	12.2	104.7 ± 7.8	7.5
PNU-142300	10	96.2 ± 3.1	3.2	105.7 ± 3.6	3.4
	80	96.8 ± 3.7	3.9	102.0 ± 2.1	2.1

#### Stability

Different experiments of stability were conducted to survey whether linezolid and PNU-142300 were still stable under many different conditions in human serum. As listed in Table 3, it was found to be stable in a routine laboratory when serum linezolid and PNU-142300 samples were placed at room temperature for 4 h, in the autosampler (10°C) for 8 h, three complete freeze (−40°C)/thaw (RT) cycles and at −40°C for at least 3 weeks.

**TABLE 3:** Stability results of linezolid and PNU-142300 in serum under different conditions (*n* = 5).

Analyte	Concentration (µg/ml)	Room temperature, 4 h		Autosampler 10°C, 8 h		Three freeze-thaw		−40°C, 3 weeks	
		RSD (%)	RE (%)	RSD (%)	RE (%)	RSD (%)	RE (%)	RSD (%)	RE (%)
Linezolid	0.02	7.0	13.1	7.6	11.8	7.5	13.2	6.7	5.8
	16	2.1	−10.3	1.8	−12.3	2.2	5.2	1.6	−12.6
PNU-142300	0.1	9.6	12.9	6.8	−13.6	8.2	−0.2	8.9	2.7
	80	3.5	8.8	3.2	−5.4	3.3	6.5	4.7	5.0

#### Application in Clinical Setting

**FIGURE 3:**
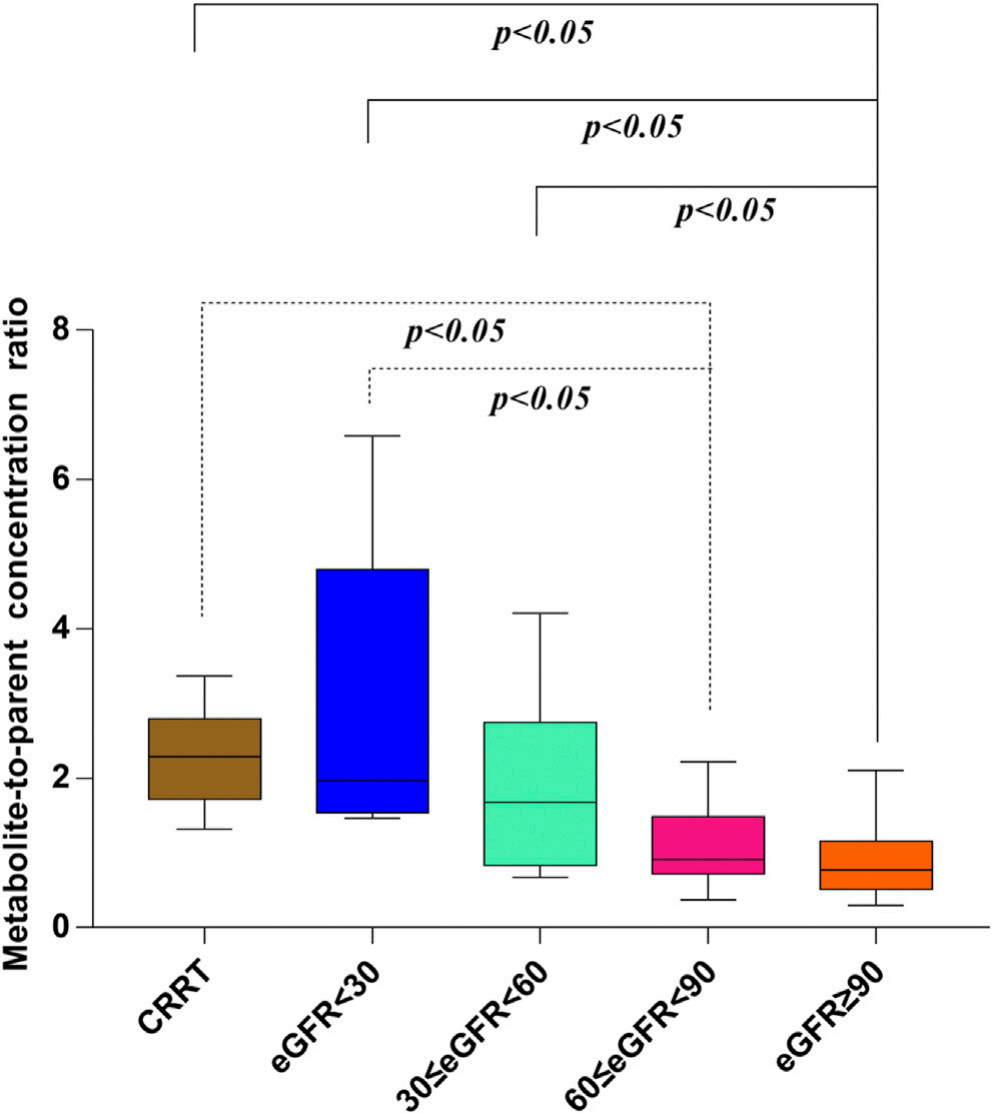
Metabolite-to-parent concentration ratios of PNU-142300 stratified by renal function. The Kruskal-Wallis test was used for comparison of metabolite-to-parent concentration ratios between groups, and *P* < 0.05 was considered as meaningful.

**FIGURE 4:**
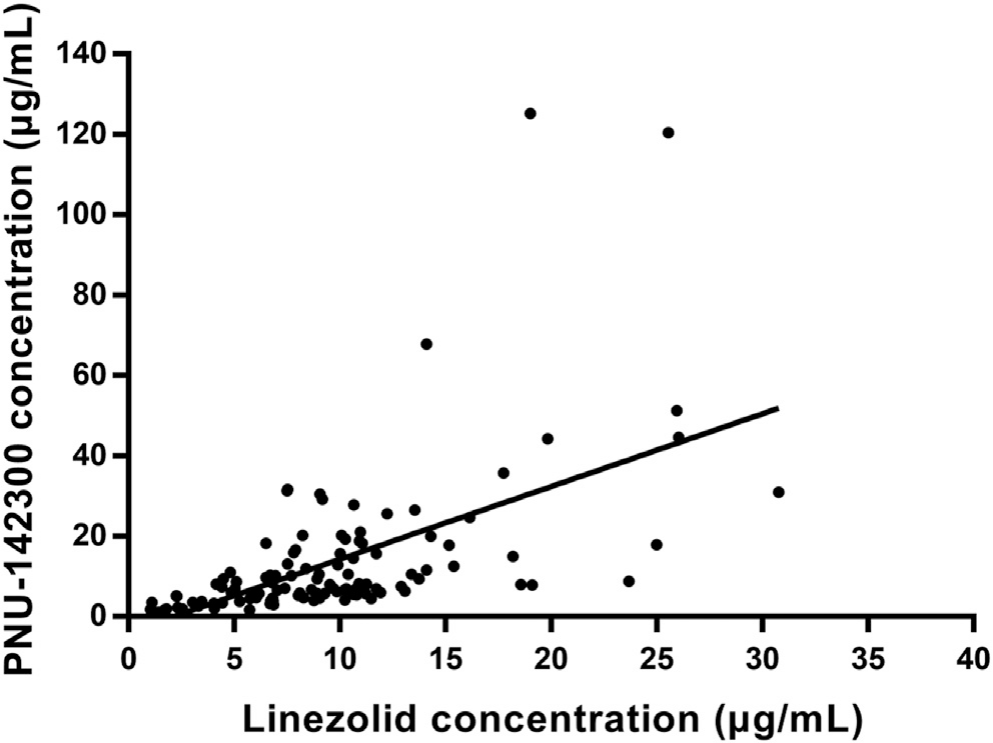
Linear regression between linezolid and PNU-142300 concentrations. (*Y* = 1.811*X—3.812, *R* ^2^ = 0.3262, indicated that PNU-142300 concentrations in paired samples were poorly correlated to linezolid concentrations).

**FIGURE 5:**
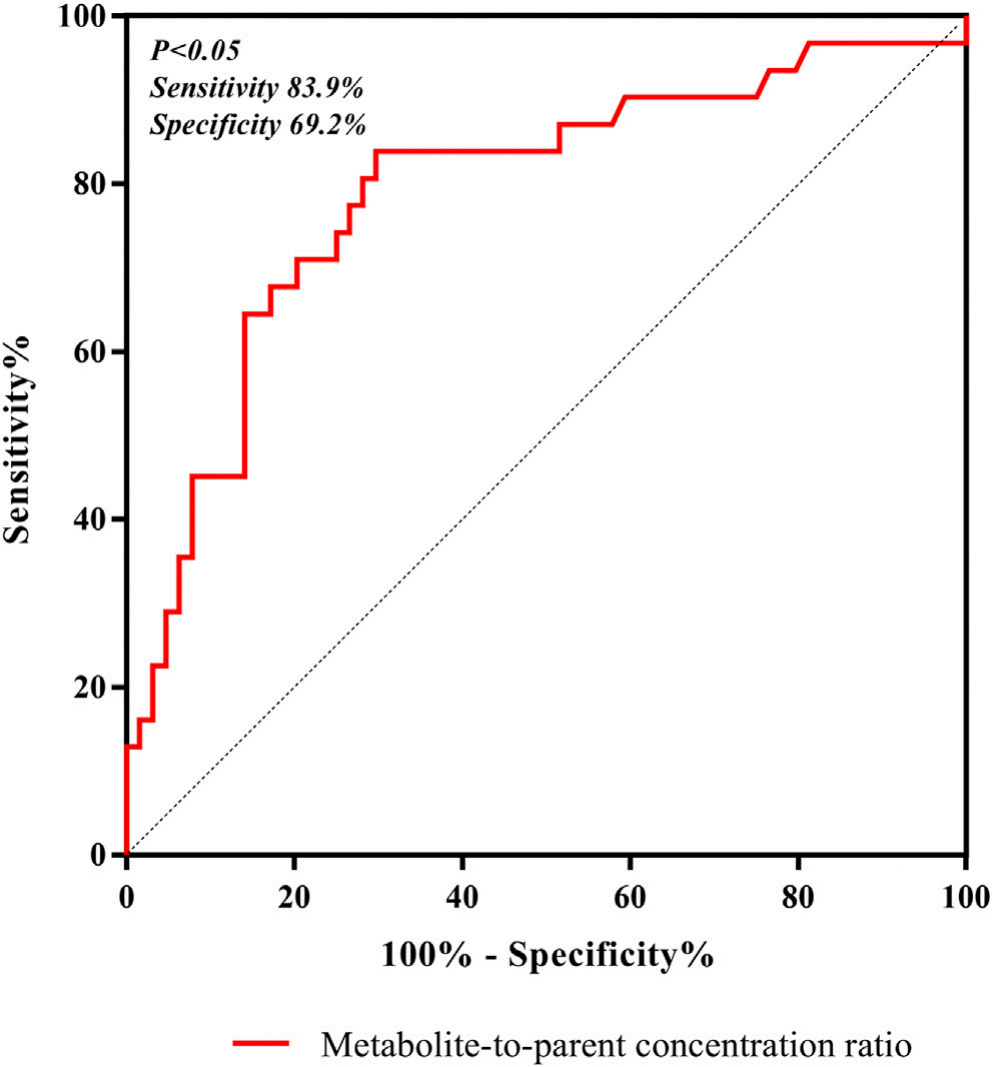
ROC curve of metabolite-to-parent concentration ratios for linezolid-associated myelosuppression, and showed that the ratio higher than 1.31 was identified as a risk factor.

In our present study, the patients were segregated into five different groups as study protocol in experimentational session. Table 4 included a summary of the clinical characteristics and demographic of the patients. The novel established UPLC-MS/MS approach was successfully performed for measuring the serum concentrations of linezolid and its one metabolite PNU-142300 in patients, and the serum concentrations of linezolid and PNU-142300 in the median (IQR) were also provided in Table 4. Compared to eGFR ≥ 90 ml/min/1.73 m^2^ in Group A, median linezolid concentrations of Group B, Group C, Group D and Group E were respectively 1.36-fold, 1.65-fold, 2.19-fold, and 1.13-fold higher, and median PNU-142300 concentrations were respectively increased 1.72-fold, 3.85-fold, 7.32-fold, and 3.87-fold. In addition, the metabolite-to-parent concentration ratios of PNU-142300 was illustrated in Figure 3, and the ratios in the renal insufficiency groups (Group C, Group D and Group E) were significantly higher than those of patients in Group A without renal impairment (*P*< 0.05) and patients in Group B with mild impairment (*P* < 0.05, except Group C). The linear relationship between linezolid and PNU-142300 concentration level was demonstrated in Figure 4 (*Y* = 1.811*X—3.812, *R* ^2^ = 0.3262), which indicated that the correlation was poor. The incidence of linezolid-associated myelosuppression was also shown in Table 4. Receiver operating characteristic (ROC) curve of metabolite-to-parent concentration ratios for linezolid-associated myelosuppression was performed in Figure 5, and the results showed that the ratio higher than 1.31 was identified as a risk factor.

**TABLE 4:** Patient characteristics and the concentrations of linezolid and PNU-142300 stratified by renal function.

Parameters	Total	eGFR ≥ 90 (ml/min/1.73 m^2^)	60 ≤ eGFR < 90 (ml/min/1.73 m^2^)	30 ≤ eGFR < 60 (ml/min/1.73 m^2^)	eGFR < 30(ml/min/1.73 m^2^)	CRRT (ESRD)
Weight (kg)	60.0 (54.0–66.0)	60.0 (53.0–67.0)	60.3 (59.0–65.3)	60.0 (52.3–63.8)	70.0 (60.0–72.0)	55.0 (43.5–66.3)
Age (years)	65.0 (54.0–77.5)	56.0 (48.0–66.0)	74.0 (68.0–81.0)	72.0 (60.8–82.3)	64.0 (52.0–80.0)	71.5 (60.8–77.3)
eGFR (ml/min/1.73 m^2^)	87.0 (54.0–106.0)	106.0 (98.8–115.0)	76.0 (68.3–83.8)	51.5 (45.0–54.3)	24.0 (17.3–25.0)	22.0 (9.0–29.0)
Sex (males/females)	64/25	31/8	14/9	9/5	6/1	4/2
Number of serum samples	115	54	28	18	8	7
Linezolid concentration (µg/ml)	8.9 (5.8–11.5)	6.9 (4.1–10.4)	9.4 (7.1–11.0)	11.4 (7.5–19.9)	15.1 (7.7–19.6)	7.8 (6.5–9.1)
PNU-142300 concentration (µg/ml)	7.5 (4.7–15.7)	4.7 (3.1–7.1)	8.1 (6.0–11.9)	18.1 (10.0–31.1)	34.4 (11.6–107.4)	18.2 (11.0–20.2)
Metabolite-to-parent concentration ratio	1.0 (0.7–1.7)	0.8 (0.5–1.2)	0.9 (0.7–1.5)	1.7 (0.8–2.8)	2.0 (1.5–4.8)	2.3 (1.7–2.8)
Time since last dose (hours)	12.4 (10.7–13.6)	12.2 (10.6–13.4)	12.1 (11.0–13.2)	13.4 (10.9–13.9)	12.0 (10.3–13.3)	12.3 (10.1–13.5)
Myelosuppression (occurrence/no occurrence)	26/63	7/32	5/18	5/9	4/3	5/1

Data are presented as median (interquartile range).

Although it has been reported that the relative clearance rate of linezolid was not affected by renal function, and no dosage adjustments have been recommended, the concentrations of the metabolites in patients with severe renal insufficiency have been estimated to be 7-8 fold than those of patients with normal renal function (Stalker and Jungbluth, 2003). Thus, it could be inferred that these metabolites may play an important role in the increased linezolid-associated myelosuppression found in patients with renal insufficiency (defined as an eGFR < 60). Recently, Pai et al. developed and validated the first bioanalytical assay for simultaneous measurement of linezolid and its major metabolites in human serum by liquid chromatography-tandem mass spectrometry (LC-MS/MS) (Souza et al., 2020b). Moreover, they found that the major metabolites can accumulate in patients with renal impairment, and the concentration levels of linezolid cannot replace the position of linezolid metabolite concentration levels (Souza et al., 2020a). However, patients in the above mentioned report were only separated into two groups with normal renal function (eGFR ≥ 60 ml/min/1.73 m^2^) and renal impairment (eGFR < 60 ml/min/1.73 m^2^), without detailed grouping and CRRT patients. Although the metabolite-to-parent ratios in patients without renal insufficiency were significantly lower than that in the group of patients with renal impairment, the trends of the metabolite-to-parent ratios had not been studied in details even when eGFR < 60 ml/min/1.73 m^2^. Thus, in our present experiment, the patients were grouped more detailed, even including CRRT patients.

From the results of our study, we confirmed that the quality of renal function played a key role in the metabolic clearance of linezolid and its metabolite PNU-142300, and the accumulation of PNU-142300 in the gradual deterioration of renal function was more obvious. A clear relationship had been identified for the exposure of PNU-142300 in patients with renal impairment. In addition, it had been found that a poor correlation existed between the concentration levels of linezolid and PNU-142300 in paired samples, which indicated that the concentration levels of linezolid in serum samples can not be used as a surrogate indicator to predict the concentration levels of PNU-142300 as reported by Pai et al. (Souza et al., 2020a). Moreover, the metabolite-to-parent concentration ratios of PNU-142300 was increased with the gradual deterioration of renal function.

Both renal impairment and linezolid exposure were closely related with the increased risk of linezolid-associated myelosuppression (Boak et al., 2014; Crass et al., 2019). However, Pai et al. did not elaborate the specific contribution of these two metabolites to linezolid-associated myelosuppression in patients (Souza et al., 2020a). From Table 4, the incidence of linezolid-associated myelosuppression was the highest in CRRT patients. However, this phenomenon cannot be explained by linezolid concentration, even its metabolite PNU-142300. Given the pharmacokinetic properties of linezolid, such as low protein binding, low molecular weight, and relatively large volume of distribution, it may potentially be removed by CRRT, and CRRT treatment dose, modalities and membrane material are the three influencing factors that associated with extracorporeal clearance by CRRT (Ide et al., 2018). In previous literature, it was also proved to be that linezolid exhibited wide differences in pharmacokinetics properties from critically ill patients after renal replacement therapy (Roger et al., 2016). These reasons may explain why linezolid concentrations decreased in CRRT patients. As we known, linezolid and its main metabolite PNU-142300 have a common chemical structure, so we think that PNU-142300 may also be removed by CRRT. Therefore, the metabolite-to-parent concentration ratios of PNU-142300 was increased in CRRT patients. In our study, we used ROC curve to assess the predictive power of the metabolite-to-parent concentration ratios of PNU-142300, and the risk threshold for linezolid-associated myelosuppression was identified. After the ROC curve analysis, the area under the ROC curve was 0.816, the maximum Youden index was 0.613, and the best optimal cut-off point was 1.31 on the ROC curve, which was prone to linezolid-associated myelosuppression. If the ratio is more than 1.31, it may illustrate that patients may have a higher risk of linezolid-associated myelosuppression, and the hematological monitoring and management of the patients should be strengthened immediately, and the dosage regimens should be adjusted according to the monitoring results. Thus, our study suggested that an elevated metabolite-to-parent concentration ratio of PNU-142300 is a risk factor for the onset of linezolid-associated myelosuppression for the first time.

Our research has a few limitations in this study. First, the nature of retrospective analysis may largely limit these conclusions. Second, the limited number of patients greatly weakened the results of the statistical comparisons among different groups according to renal function and the groups were not strictly matched in this present experiment, but the actual changes in patients with infection during linezolid treatment were reflected by the observed differences in the clinical parameters. Third, our current experiment was not involved another metabolite PNU-142586 for measurement and its contribution to linezolid-associated myelosuppression, and further prospective research should pay more attention to this issue.

## Conclusion

In conclusions, a robust, quick and accurate UPLC-MS/MS assay was fully optimized and developed to detect the serum concentration levels of linezolid and PNU-142300 in subjects. This optimized method offered significant advantages according to short analysis time (only 3.0 min) and cost-effective sample preparation (a rapid and simple protein precipitation with acetonitrile). The applicability of the optimized UPLC-MS/MS approach was shown in a high-throughput routine TDM from real-world clinical practice. And we found that linezolid-associated myelosuppression occurs more frequently in patients with renal impairment, and the metabolite-to-parent concentration ratio of PNU-142300 is predicted to reduce this toxicity of the treatment.

## Data Availability

The original contributions presented in the study are included in the article/Supplementary Material, further inquiries can be directed to the corresponding authors.
